# Intensive multidisciplinary rehabilitation modulates serum *α*-synuclein and miRNA expression in Parkinson’s disease: preliminary results of a randomized study

**DOI:** 10.3389/fncel.2026.1760593

**Published:** 2026-05-28

**Authors:** Simone Agostini, Mario Meloni, Roberta Mancuso, Riccardo Nuzzi, Giulia Ferraro, Anna Salvatore, Pietro Arcuri, Anna Castagna, Jorge Navarro, Francesca Lea Saibene, Mario Clerici

**Affiliations:** 1 IRCCS Fondazione Don Carlo Gnocchi, Milan, Italy; 2 Neurology Unit, Azienda Ospedaliero-Universitaria, Cagliari, Italy; 3 Department of Pathophysiology and Transplantation, University of Milan, Milan, Italy

**Keywords:** biomarkers, brain-derived neurotrophic factor (BDNF), intensive outpatient multidisciplinary rehabilitation program, miRNAs, Parkinson’s disease, randomized, single-blind, controlled, two-arm trial

## Abstract

**Introduction:**

Parkinson’s disease is a progressive neurodegenerative condition, and unfortunately, there are currently no treatments available that can halt or slow down its progression. However, recent research on intensive and multidisciplinary rehabilitation shows great promise, indicating that vigorous exercise may offer benefits to patients. This study is a randomized, single-blind, controlled, two-arm trial comparing an intensive outpatient multidisciplinary rehabilitation program with a home-based self-administered stretching program in Persons with Parkinson’s disease (PwPD). This study aims to assess the effects of an intensive rehabilitation program compared to a home-based self-treatment plan on the serum expression of four molecular biomarkers: brain-derived neurotrophic factor (BDNF), total *α*-synuclein, miR-223-3p, and miR-7-1-5p in PwPD.

**Methods:**

We enrolled seventy PwPD in mild-to-moderate stages (average age: 71.15 ± 16.19 years, disease duration: 7.67 ± 5.61 years; UPDRS: 38.06 ± 13.07). Out of these, 36 participants (19 males and 17 females) were assigned to an outpatient daily intensive multidisciplinary rehabilitation treatment (EXP-PwPD), while 34 (22 males and 12 females) participated in a home-based self-treatment stretching program (CTRL-PwPD). Serum samples were taken at baseline (T0), at the end of the intervention period (T1, 6 weeks after T0), and at T2 (3 months after T1). We measured the protein concentrations (BDNF and *α*-synuclein) and circulating miRNAs (miR-223-3p and miR-7-1-5p) in the serum, and compared results between the groups.

**Results and discussion:**

The intensive rehabilitation program led to a significant increase in serum BDNF and *α*-synuclein expression. Notably, the rise in serum α-synuclein was dependent on exercise, returning to baseline concentration at T2, while serum BDNF remained significantly elevated even 2 months post-program completion (T2) (*p* = 0.03). Among the miRNAs studied, miR-7-1-5p reflected the pattern of BDNF, showing a notable increase after intensive rehabilitation (*p* = 0.05) and persistence at follow-up (*p* = 0.04). There were no significant changes for miR-223-3p. In conclusion, this study indicates that intensive rehabilitation can modify circulating biomarkers in PD. Our findings indicate that intensive exercise induces a sustained elevation of serum BDNF and miR-7-1-5p, reflecting enhanced neuroplastic and neurotrophic activity.

**Clinical trial registration:**

ClinicalTrials.gov, identifier: NCT05452655.

## Introduction

Parkinson’s disease (PD), one of the most common neurodegenerative diseases, is a chronic disorder affecting movement which is characterized by resting tremor, rigidity and bradykinesia (Beitz, 2014). The main neuropathological feature of PD is the accumulation of misfolded *α*-synuclein (α-syn) in substantia nigra of the brain (Beitz, 2014). This contributes to the neuroinflammatory status of the disease (de Araujo et al., 2022), with damage and loss of dopaminergic neurons leading to a reduction of movement ability (Poewe et al., 2017). Brain-derived neurotrophic factor (BDNF) has attracted considerable attention as a critical regulator of neuronal survival, differentiation, and synaptic plasticity (Scalzo et al., 2010). Reduced BDNF concentrations have been reported in the substantia nigra of PD animal models (Baquet et al., 2005), as well as in post-mortem PD brains (Howells et al., 2000), supporting its potential role in disease pathogenesis and neurorestorative mechanisms. Like most proteins, BDNF and *α*-syn expression is modulated by micro ribonucleic acids (miRNAs), short non-coding single-stranded RNAs that play a crucial role in the modulation of gene expression by binding to the 3’untranslated region (3’UTR) of their mRNA targets (Lu and Rothenberg, 2018). The expression of BDNF is regulated, among others, by miR-7-1-5p (Li et al., 2019), whereas *α*-syn by both miR-7-1-5p (Doxakis, 2010; Choi et al., 2018) and miR-223-3p (Cressatti et al., 2019). Our recent studies (Mancuso et al., 2019; Citterio et al., 2025) showed that miR-7-1-5p and miR-223-3p are significantly higher in serum of persons with PD (PwPD) compared to control subjects, suggesting that these miRNAs may serve as circulating markers of PD-related molecular pathways and post-transcriptional regulation of neuroplasticity and synapse-related proteins.

Motor impairments represent one of the most disabling aspects for PwPD, severely affecting independence and quality of life. Although dopaminergic treatments, including levodopa and dopamine agonists, primarily provide symptomatic relief of motor symptoms in PD (Connolly and Lang, 2014), a substantial proportion of PwPD continues to experience residual motor deficits despite optimized medical therapy (Sprenger and Poewe, 2013). Consequently, non-pharmacological interventions, particularly exercise and rehabilitation, have gained increasing attention as complementary strategies to improve functional outcomes (Sharpe et al., 2020). Several studies indicate that multidisciplinary rehabilitation may be more effective than conventional or home-based approaches in improving quality of life, motor and non-motor symptoms, and functional outcomes in PwPD. These benefits are attributed to the structured, supervised, and high-intensity integration of aerobic, motor, and cognitive training, which characterizes multidisciplinary intensive programs (Ferrazzoli et al., 2018; Frazzitta et al., 2015; Lo Buono et al., 2021; Meloni et al., 2021).

Beyond its well-established effects on motor and non-motor symptoms, intensive rehabilitation is increasingly recognized as being a potent modulator of neuroplasticity-related pathways in PD (Rotondo et al., 2023). Among the molecular mediators implicated in activity-dependent neural adaptation, BDNF plays a central role in synaptic plasticity, neuronal survival, and learning, and its circulating levels have consistently been shown to increase in response to physical exercise, particularly following high-intensity training (Frazzitta et al., 2014). Similarly, *α*-syn, while classically associated with PD pathology, is a key presynaptic protein involved in synaptic-vesicle dynamics and neuronal activity, and its circulating levels appear to be modulated by exercise-induced neuronal activation (Zhou et al., 2017; Dutta et al., 2022).

Intensive multidisciplinary rehabilitation programs produce greater and more sustained motor benefits compared to conventional or low-intensity interventions, suggesting that training intensity may be a critical determinant of neural adaptation in PD. However, despite these clinical observations, the biological mechanisms underlying these benefits remain incompletely understood. In particular, it remains unclear whether intensive rehabilitation can induce sustained changes in circulating biomarkers of neuroplasticity in humans with PD, and whether such changes are associated with modulation of their post-transcriptional regulatory miRNAs.

Building on this evidence, we developed a 6-week intensive multidisciplinary rehabilitation protocol aimed at providing a structured and clinically supervised alternative to standard home-based self-administered programs (Saibene et al., 2024). Within the framework of a randomized controlled trial, the primary aim of the present study is to assess the effects of an intensive versus home-based rehabilitation program on the expression of serum biomarkers. Specifically, we evaluate change in circulating BDNF and total *α*-syn expression, and further investigate whether exercise-induced modulation of these proteins may be associated with changes in their post-transcriptional regulatory miRNAs, including miR-7-1-5p and miR-223-3p, both during the intervention and 3 months after the end of the treatment.

## Materials and methods

### Patients

A total of 70 PwPD (41 male and 29 female, mean age 71.15 ± 16.19 years) were consecutively recruited at the Center for Diagnosis and Rehabilitation of Parkinson’s Disease and Parkinsonisms (DiaRiaPARK) of the IRCCS Fondazione Don Carlo Gnocchi in Milan, Italy. DiaRiaPARK is a specialized tertiary outpatient center dedicated to the diagnosis and multidisciplinary rehabilitation of PD and atypical parkinsonisms. All participants were outpatients at the time of enrollment.

The diagnosis of PD was made in accordance with the Movement Disorder Society (MDS) Clinical Criteria for PD (Postuma et al., 2015). After enrollment, participants were randomly allocated to either an intensive outpatient multidisciplinary rehabilitation program (Experimental group, EXP-PwPD; *n* = 36), or a home-based self-treatment stretching program (CTRL-PwPD, *n* = 34) in a 1:1 ratio using a computer-generated block randomization procedure, stratified according to disease severity based on the modified Hoehn and Yahr stage (mH&Y = 1.5 to 3). Both interventions were delivered over six consecutive weeks, 5 days per week (Monday to Friday), for a total of 30 treatment days. The study followed a single-blind design. Outcome assessors, including the movement disorder specialist responsible for clinical evaluations, were blinded to group allocation. Laboratory personnel performing biomarker analyses were also blinded to group assignment. Due to the nature of the interventions, participants and treating therapists could not be blinded. However, participants were not informed about the expected benefits of either intervention or the study hypothesis in order to minimize expectation bias.

Motor training consisted of twice-daily physiotherapy sessions (40 min each) delivered within an intensive outpatient framework and based on evidence-based physiotherapy recommendations for PD. A task-oriented circuit training component combining treadmill aerobic training and balance-oriented, task-specific exercises was delivered three times per week on alternate days, as previously described. During treadmill training, exercise intensity was targeted at moderate-to-high effort (Rate of Perceived Exertion, Borg 6–20 scale: 13–15), with progressive adjustment of speed and incline. The remaining sessions were individualized according to clinical needs (e.g., resistance training, dual-task practice, cueing strategies/freezing-of-gait management, joint mobilization, and biofeedback-based exercises).” (Keus et al., 2013; Saibene et al., 2024). Cognitive rehabilitation was individualized and supervised by a neuropsychologist and targeted executive, attentional, visuospatial, memory, and language functions. Treatment was delivered both in a standard format (40 min, three times/week) and using a semi-immersive Virtual Reality Rehabilitation System (VRRS) (60 min, twice/week), consisting of structured computerized tasks with progressive difficulty and performance feedback (Manenti et al., 2020).

To support compliance in the PwPD-CTRL group, participants received standardized written instructions and a supervised in-person training session prior to starting the home program. Adherence and fidelity were remotely monitored using a structured daily diary in which participants recorded completion of the prescribed stretching/mobilization exercises, any side effects, and any difficulties encountered. Diaries were collected and reviewed by the clinical staff at the end of the 6-week period; adherence was operationalized as the number of completed sessions over the 30 prescribed sessions (Monday–Friday for 6 weeks). No real-time supervision or feedback was provided during home sessions. The home-based control intervention was intentionally designed as a low-intensity, non-aerobic program to act as an active control condition, allowing participant to remain engaged in a perceived therapeutic activity while minimizing the activation of exercise-related biological pathways potentially influencing the investigated biomarkers.

To minimize the risk of contamination between the experimental and control groups, several procedural measures were implemented. Participants allocated to the EXP-PwPD group attended the intensive multidisciplinary rehabilitation program exclusively within the outpatient clinical setting, whereas participants allocated to the CTRL-PwPD group performed a home-based self-administered program and did not attend the rehabilitation facility during the intervention period. The two interventions were delivered in different settings and involved different levels of supervision and contact with the rehabilitation staff. Participants were instructed not to modify their usual physical activity routines outside the assigned intervention and not to engage in additional structured physical activity outside the assigned intervention during the study period. No specific dietary restrictions were imposed, in order to maintain real-world conditions. Group allocation was not disclosed to participants in terms of expected benefits, reducing the likelihood of cross-group behavioral adaptation.

**Figure 1:**
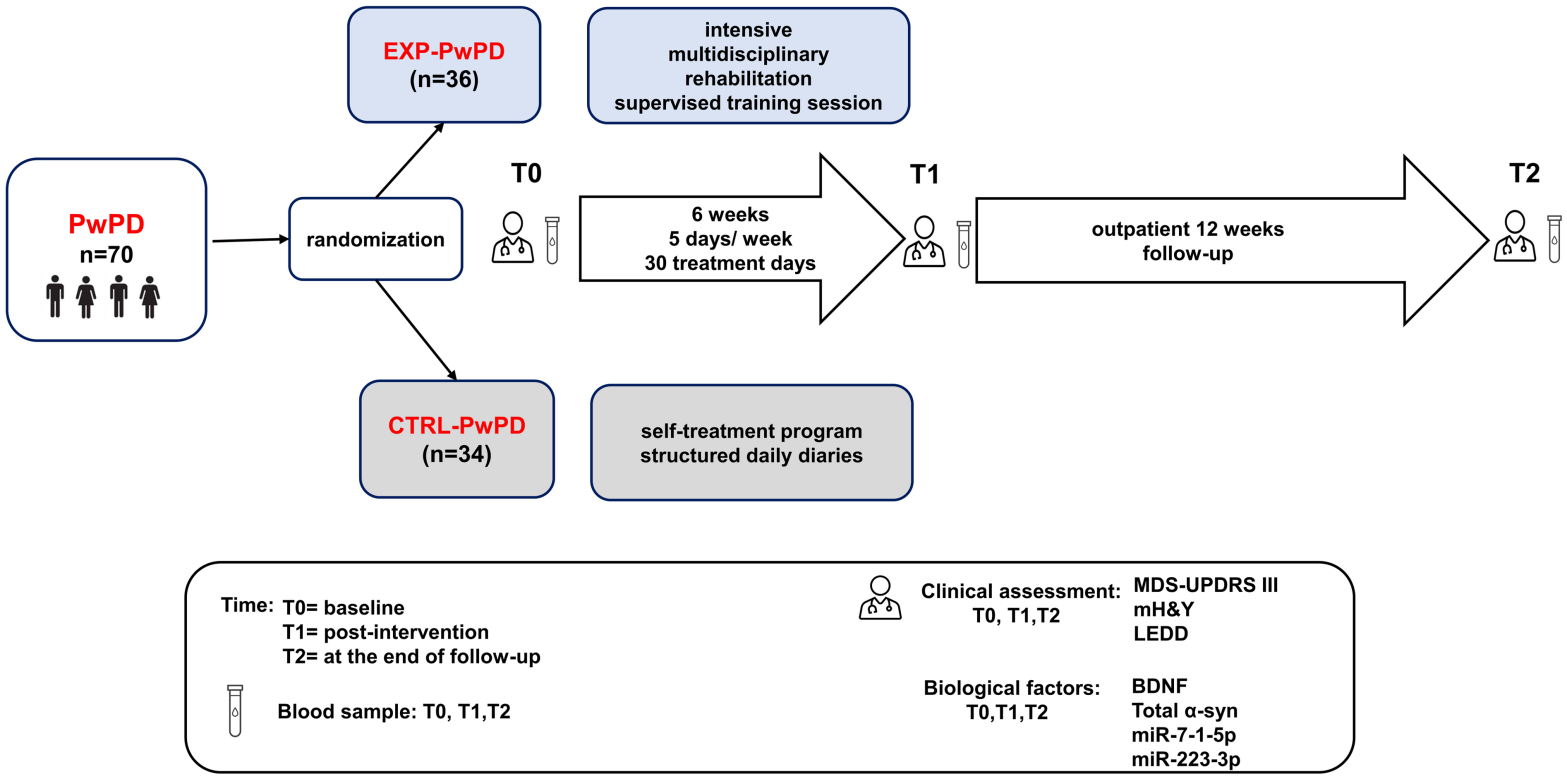
Schematic representation of the study design. PwPD, Persons with Parkinson’s disease; EXP-PwPD, Experimental group; CTRL-PwPD, control group, MDS-UPDRS-III, Movement disorder society—Unified Parkinson’s disease Rating Scale part III; LEDD, Levodopa equivalent daily dosage; mH&Y, Modified Hoehn and Yahr scale; BDNF, Brain-Derived Neurotrophic Factor; α-syn, Alpha synuclein; miR, miRNA.

Peripheral blood samples were collected in the morning under standardized conditions to minimize circadian variability from each PwPD, via venipuncture of the antecubital region, in accordance with standard clinical procedures, at three predefined time points: before the start of the rehabilitation program (T0), immediately after completion of the 6-week intervention (T1), and 12 weeks following the end of the program (T2). At T0, blood was drawn approximately 1 hour before the start of the rehabilitation program. At T1, blood was collected on the morning of the final treatment day, and at T2, blood was drawn on the morning of the 12th week after treatment completion. Blood collections was always performed while participants were in the on dopaminergic medication state. Figure 1 schematizes the experimental design.

No specific instructions regarding fasting, smoking, or alcohol intake were imposed beyond standard clinical recommendations. Participants were asked to avoid strenuous physical activity on the morning of blood collection. Blood samples were collected by nurses who were not involved in the study and all laboratory analyses were performed by biologists who was blinded to group allocation. Both sample collection and biomarker analysis were conducted under blinded conditions to minimize potential bias.

Serum was obtained by centrifugation (2000 x *g* for 10′ at room temperature) and stored at −80 °C; absence of hemolysis was assessed by visual inspection and by spectrophotometric measurement of hemoglobin absorbance at 414 nm (Shah et al., 2016).

The study was conducted in accordance with the ethical principles of the Declaration of Helsinki. All subjects gave informed and written consent according to a protocol approved by the local ethics committee of the IRCCS Don Carlo Gnocchi Foundation (project identification code 1_16/04/2020, and subsequent amendments identified with ID 8/2021/CE_FdG/FC/SA and with ID 13/2023/CE_FdG/FC/SA).

Compliance with the rehabilitation programs was assessed differently for the two groups. In the EXP-PwPD group, attendance was recorded daily by the rehabilitation staff. In the CTRL-PwPD group, adherence was monitored using structured daily diaries in which participants recorded completion of the prescribed home exercises. Four PwPD (2 in EXP-PwPD and 2 in CTRL-PwPD group) drop-out the study after T1. MDS-UPDRS characterization of 1 PwPD at T0, 4 at T1 and 3 at T2 were missed.

### Clinical assessments

Participants with PD underwent a comprehensive clinical assessment, including motor evaluation using the Movement Disorder Society-sponsored revision of the Unified Parkinson’s Disease Rating Scale Part III (MDS-UPDRS III) (Goetz et al., 2008). Disease severity was staged according to the Modified Hoehn and Yahr Scale (mH&Y) (Hoehn and Yahr, 1967; Goetz et al., 2003). Levodopa Equivalent Daily Dose (LEDD) was calculated according to established conversion factors (Tomlinson et al., 2010). Clinical relevance of changes in motor performance was interpreted using the Minimal Clinically Important Difference (MCID) for the MDS-UPDRS Part III. Based on established anchor-based estimates, a decrease of 3.25 points was considered the threshold for minimal clinically important improvement, whereas an increase of 4.63 points was considered the threshold for minimal clinically important worsening (Horvath et al., 2015).

### Serum miRNA extraction, cDNA reverse transcription and quantification

miRNAs were extracted from 200 μL of serum using spin column-based kit (miRNeasy Mini kit, Qiagen, Hilden, Germany) by QIAcube connect (Qiagen). miRNAs were retrotranscribed in cDNA by miRCURY LNA RT Kit (Qiagen) in a total volume of 10 μL, as specified in the manufacturer’s instruction. The absolute quantification of miR-7-1-5p (Qiagen assay number: YP00205877 and miR-223-3p (Qiagen assay number: YP00205986) was performed by droplet digital PCR (ddPCR QX200, Bio-Rad, Hercules, CA, US). In particular, 3 μL of diluted cDNA (1,10 for miR-7-1-5p and 1:25 for miR-223-3p) were mixed with ddPCR EvaGreen Supermix (Bio-Rad), LNA™ specific primers (Qiagen), and then emulsified with droplet generator oil (Bio-Rad) by QX200 droplet generator (Bio-Rad). Droplets were transferred to a 96-well reaction plate and heat-sealed with a pierceable foil sheet by a PCR plate sealer (PX1, Bio-Rad). PCR amplification was performed in sealed 96-well plate using a T100 thermal cycler (Bio-Rad) as follows: 10′ at 95 °C, 40 cycles at 94 °C for 30″ and 58 °C for 60″, followed by 10′ at 98 °C and a hold at 4 °C. The plate was then transferred to a QX200 droplet reader (Bio-Rad). Each well was queried for fluorescence to determine the quantity of positive events (droplets), and the results were displayed as dot plots The miRNA concentration was expressed as copies/ng of extracted RNA.

### Proteins measurement

Brain-Derived Neurotrophic Factor (BDNF) was measured from 100 μL of diluted serum (1:100) for each time point by commercial Enzyme-Linked ImmunoSorbent Assays (ELISA), according to the manufacturer’s instructions (DBA Italia, Segrate, Milan, Italy). Briefly, 100 μL of diluted serum (dilution 1:100) were transferred into the pre-coated microwells and the plate was incubated for 1 h at 37 °C. After liquid removal, 100 μL of detection reagent A was added in each well, and incubated for 1 h at 37 °C. After washing steps, 100 μL of detection reagent B was added in each well, and incubated for 30 min at 37 °C. After washing steps, 90 μL of substrate solution was added in each well, and incubated for 15 min at 37 °C protected from light. Finally, 50 μL of stop solution was added in each well and the reaction stopped.

Soluble total *α*-syn (ng/mL) was measured from 100 μL of diluted serum (dilution: 1:2) for each time point by commercial Enzyme-Linked ImmunoSorbent Assays (ELISA) according to the manufacturer’s instructions (IBL International, Hamburg, Germany) and as previously described (Agostini et al., 2021).

Both plates (for BDNF and for α-syn detection) were read on a plate reader (Sunrise, Tecan, Mannedorf, Switzerland) within 30 min from the addition of stop solution and optical densities (OD) of wells were determined at 450 nm; both proteins were expressed as ng/mL. The detection limits are 0.061 ng/mL for BDNF, and 0.03 ng/mL for α-syn.

### Statistical analysis

To monitor the effect of rehabilitation on the serum expression of total α-synuclein, BDNF, miR-7-1-5p and miR-223-3p in PwPD, the variation (*Δ*) from baseline in concentration of these 4 biomarkers at the different time-points was calculated, as the difference between their concentration at T1 (post-treatment) compared to T0 (baseline) (ΔT1) or at T2 (follow-up) compared to T0 (baseline) (ΔT2). Statistical analyses were performed using the commercial MedCalc Statistical Software package (Version 11.5.0.0; Ostend, Belgium). Normally distributed data were summarized as mean ± standard deviation. Not-normally distributed data were summarized as median and interquartile range (IQR: 25th and 75th percentile). For within-group comparisons across time points (T0, T1, and T2), repeated measures ANOVA was applied for normally distributed data (demographic data, clinical data and laboratory data expressed as Δ values), followed by paired t-tests for post-hoc pairwise comparisons (e.g., T1 − T0, T2 − T1). For non-normally distributed data (laboratory data expressed as absolute values), the Friedman test was used for within-group comparisons, with Wilcoxon signed-rank test for pairwise post-hoc analyses. For between-group comparisons at the same time point (e.g., T0 vs. T0, T1 vs. T1, T2 vs. T2) or for *Δ* values between groups, unpaired Student’s *t*-test was used for normally distributed data, and Mann–Whitney test for non-normally distributed data. Group comparisons involving more than two groups were performed using one-way ANOVA or Kruskal–Wallis test as appropriate. Correlations were analyzed using Spearman’s correlation coefficient. For ddPCR analysis, the QX Manager software, version 1.2 (Bio-Rad) was used. Thresholds were determined manually for each experiment using a negative control (non-reverse-transcribed miRNA) and a no template control (water). Droplet positivity was determined by fluorescence intensity. Positive and negative controls were included in each experiment. Samples resulted in less than two positive droplets are considered undetectable (Agostini et al., 2020). For the statistical analysis a concentration of 0.01 copies/ng was assumed for undetectable miRNAs. Regarding biological data, the absolute values were not-normally distributed, whereas the Δ values were normally distributed, as assessed by the Shapiro–Wilk test. A mixed-design repeated-measures ANOVA was performed with Time (T0, T1, and T2) as the within-subject factor and Group (EXP-PwPD and CTRL-PwPD) as the between-subject factor to assess longitudinal changes in MDS-UPDRS Part III scores. Sphericity was evaluated with Mauchly’s test, and Greenhouse–Geisser corrections were applied when appropriate. When significant main effect or interactions were observed, Bonferroni-adjusted post-hoc pairwise comparisons were conducted to explore differences across time points. Given three planned pairwise comparisons (T0 vs. T1, T1 vs. T2, and T0 vs. T2), the Bonferroni-adjusted significance threshold was set at *p* < 0.0167. For all the statistical tests, statistical significance was set at 2-sided *p* < 0.05. Test–retest reliability for BDNF, miRNAs, and *α*-syn measures was assessed using the Intraclass Correlation Coefficient (ICC), and all values were above 0.90. Currently there are no clinical studies examining the effectiveness of rehabilitation treatment on our primary outcomes. Therefore, the sample size was determined based on the preliminary results of our recent cross-sectional study (Agliardi et al., 2019). With a sample size of 72 subjects, 0.05 alpha and a drop-out percentage of 20%, the a-priori statistical power is 80%.

## Results

### Baseline characteristics of patients

Thirty-six PwPD were included in the EXP-PwPD group (19 males and 17 females); 34 patients formed the CTRL-PwPD group (22 males and 12 females).

According to the mH&Y scale most participants were in the mild to moderate disease stage (mean mH&Y = 2.26 ± 0.52) of the disease. The mean disease duration and motor impairment (according to the MDS-UPDRS Part III) for the overall cohort were, respectively, 7.67 ± 5.61 years and 38.1 ± 13.1.

Within the EXP-PwPD, the mean disease duration was 8.42 ± 6.39 years, whereas in the CTRL-PwPD the mean disease duration was 6.88 ± 4.61 years; this difference was not statistically significant. At baseline (T0), motor impairment was also comparable between groups, with MDS-UPDRS Part III scores of 40.64 ± 12.90 in the EXP group and 35.18 ± 13.16 in the CTRL group, with no statistically significant between-group difference.

No significant baseline differences were present between groups. The significant Time × Group interaction was associated with a moderate effect size (partial η^2^ = 0.10).

Adverse events and serious adverse events were monitored throughout the intervention period in both groups. No serious adverse events related to interventions were reported. Minor and transient discomfort related to physical exercise (e.g., fatigue or muscle soreness) was rarely reported in the EXP-PwPD group and resolved spontaneously without requiring medical intervention.

Clinical and demographic characteristics of all patients are shown in Table 1.

**Table 1:** Demographic and clinical characteristics of PwPD grouped for rehabilitation program.

Parameters	EXP-PwPD	CTRL-PwPD
N	36	34
Sex (M-F)	19:17	22:12
Age, years	69.81 ± 5.27	69.68 ± 7.13
Disease duration, years	8.42 ± 6.39	6.88 ± 4.61
MDS-UPDRS III T0	40.69 ± 12.59	35.18 ± 13.16
MDS-UPDRS III T1	34.17 ± 12.98	35.77 ± 12.98
MDS-UPDRS III T2	40.64 ± 12.90	38.01 ± 13.76
LEDD, mg/die T0	722.09 ± 343.45	633.85 ± 361.25
LEDD, mg/die T1	722.09 ± 343.45	633.85 ± 361.25
LEDD, mg/die T2	708.41 ± 341.62	633.85 ± 361.25
mH&Y stages T0	2.37 ± 0.50	2.13 ± 0.53
mH&Y stages T1	2.36 ± 0.47	2.20 ± 0.56
mH&Y stages T2	2.39 ± 0.45	2.24 ± 0.57

PwPD, Persons with Parkinson’s disease; M, male; F: female; N, absolute number; SD, standard deviation; MDS-UPDRS-III, Movement disorder society—Unified Parkinson’s disease rating scale part III; LEDD, levodopa equivalent daily dosage; mH&Y, modified Hoehn and Yahr scale (Hoehn and Yahr, 1967; Goetz et al., 2003). Data are expressed as mean±SD.

### Effect of rehabilitation on clinical outcomes

The mixed-design ANOVA revealed a significant main effect of Time on motor performance [*F* (2,114) = 17.06; *p* < 0.001], indicating that MDS-UPDRS-III scores changed significantly across T0, T1, and T2. A significant Time × Group interaction was also observed [F (2,114) = 6.39; *p* = 0.002].

Post-hoc comparisons showed that the EXP-PwPD exhibited a significant reduction in MDS-UPDRS-III scores from T0 to T1 (mean change = −6.5 points), whereas no significant changes over the same interval were detected in the CTRL-PwPD. At follow-up (T2), the EXP-group returned to near-baseline values, while the CTRL-PwPD group again showed no significant longitudinal variation.

In the EXP-PwPD group, MDS-UPDRS Part III scores decreased from 40.7 at baseline (T0) to 34.1 after intensive rehabilitation (T1), corresponding to a mean change of −6.6 points. Subsequently, UPDRS-III scores increased from 34.1 at T1 to 40.6 at follow-up (T2), corresponding to a mean change of +6.5 points.

**Figure 2:**
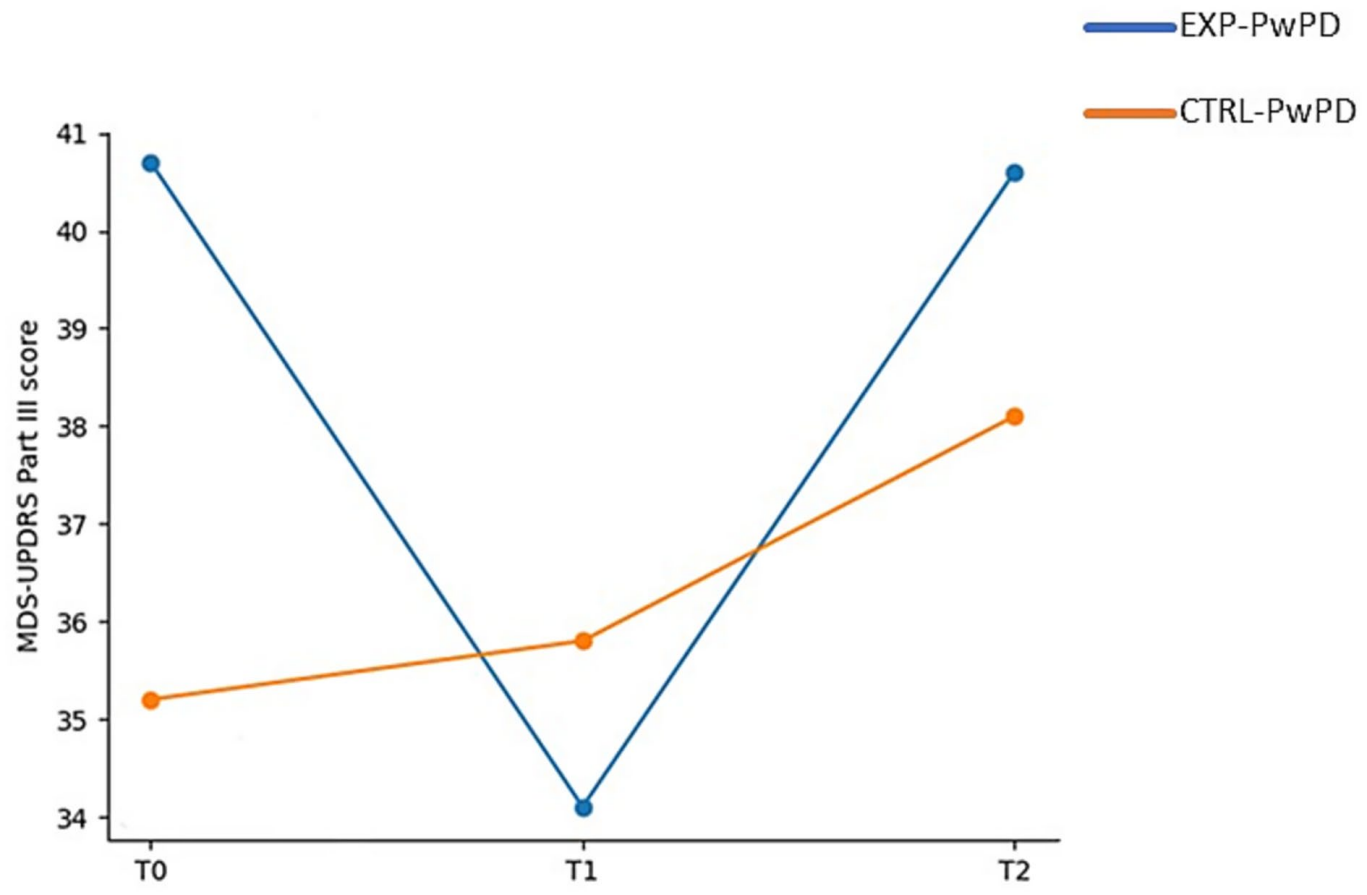
Longitudinal changes in motor performance assessed by the MDS-UPDRS Part III. Mean MDS-UPDRS Part III scores at baseline (T0), end of treatment (T1), and follow-up (T2) in the experimental (EXP-PwPD) and control (CTRL-PwPD) groups. The figure illustrates the significant main effect of Time and the Time × Group interaction observed in the mixed-design ANOVA.

The longitudinal changes in MDS-UPDRS Part III scores in the EXP-PwPD and CTRL-PwPD groups are shown in Figure 2.

### Effect of rehabilitation on biomarkers

**Figure 3:**
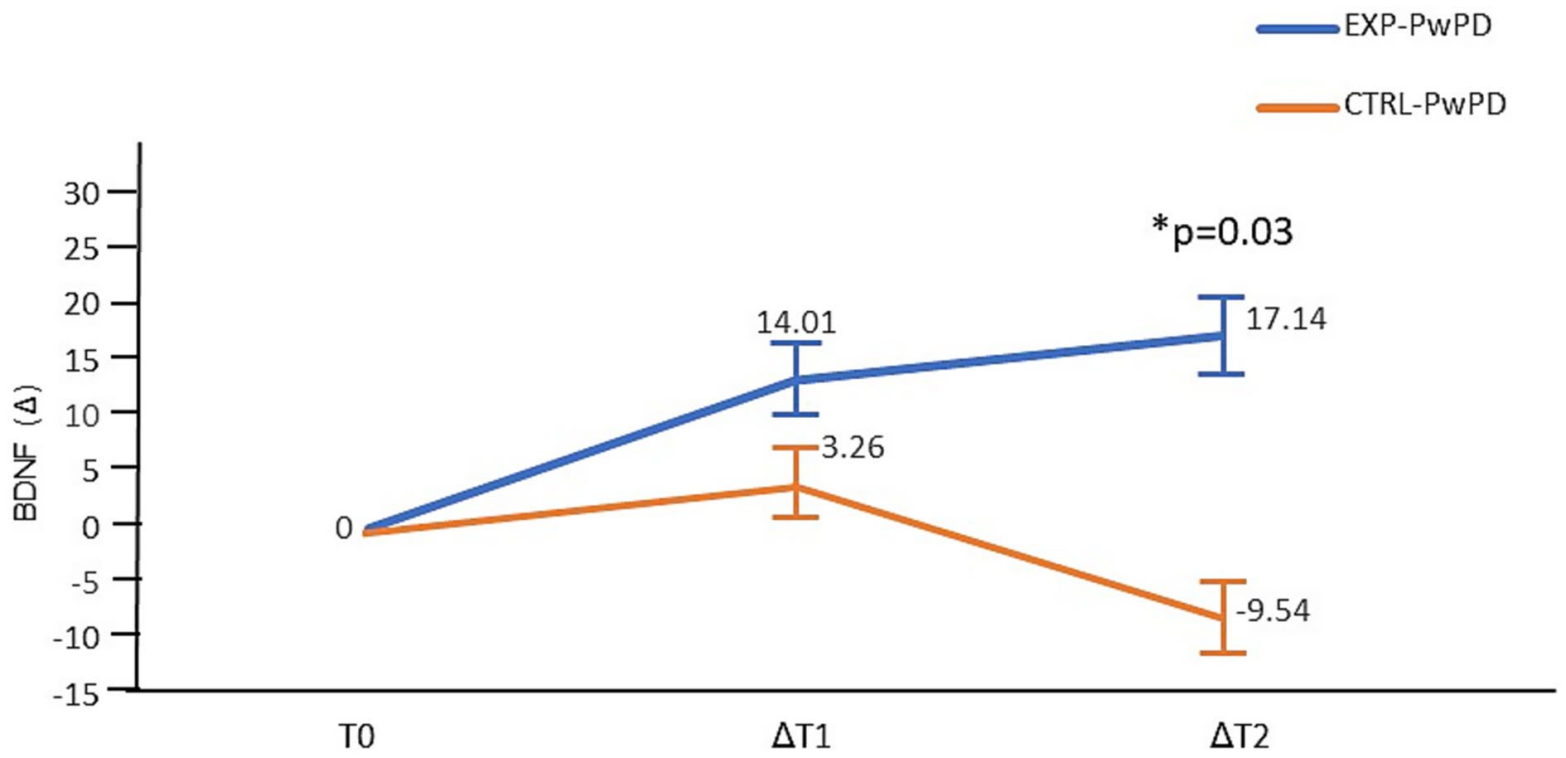
Serum BDNF concentration changes from baseline (*Δ*) at T1 (ΔT1) and T2 (ΔT2) in EXP-PwPD (blue) and CTRL-PwPD (orange) groups. Error bars represent standard deviation. *p*-value indicates the statistical difference in ΔT2 between EXP-PwPD *vs.* CTRL-PwPD (Student’s *t*-test).

As shown in Figure 3, the rehabilitation led to an overall increase in serum BDNF concentration, which was more pronounced in the EXP-PwPD group compared to the CTRL-PwPD group. In EXP-PwPD, the BDNF serum concentration showed a significant increase even 12 weeks after the end of the rehabilitation program (mean ΔT2: 17.14 ± 7.51), whereas for CTRL-PwPD, its concentration decreased after the end of rehabilitation (mean ΔT2: −9.54 ± 7.38). The between group comparison of ΔT2 values (EXP-PwPD *vs.* CTRL-PwPD) was statistically significant (*p* = 0.03).

**Figure 4:**
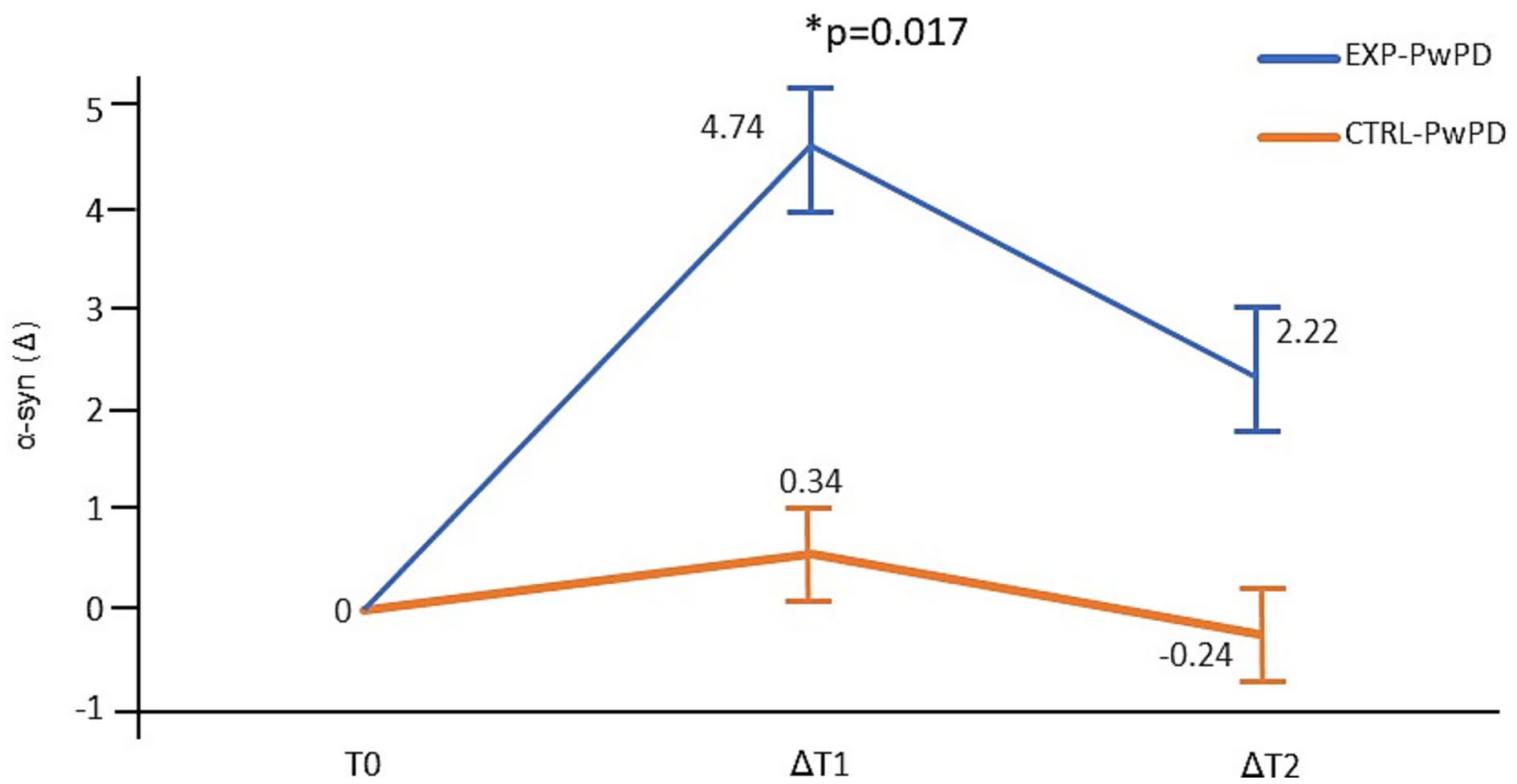
Serum α-synuclein concentration changes from baseline (Δ) at T1 (ΔT1) and T2 (ΔT2) in EXP-PwPD (blue) and CTRL-PwPD (orange) groups. Error bars represent standard deviation. *p*-value indicates the statistical difference of ΔT1 between EXP-PwPD *vs.* CTRL-PwPD (Student’s *t*-test).

Regarding total *α*-synuclein, the two rehabilitation programs showed different patterns of response. In the intensive rehabilitation group, total serum α-synuclein concentrations exhibited a significant increase after the intervention (T0: 13.30; 8.71–17.36; T1: 18.54; 15.16–22.19; *p* = 0.009), coming back to baseline values 12 weeks after the end of the program (T2), whereas in the CTRL-PwPD group, no change was observed across time points. The between-group comparison of ΔT1 *α*-synuclein revealed a statistical difference between EXP-PwPD *vs.* CTRL-PwPD: *p* = 0.017). All these results are reported in Figure 4.

**Figure 5:**
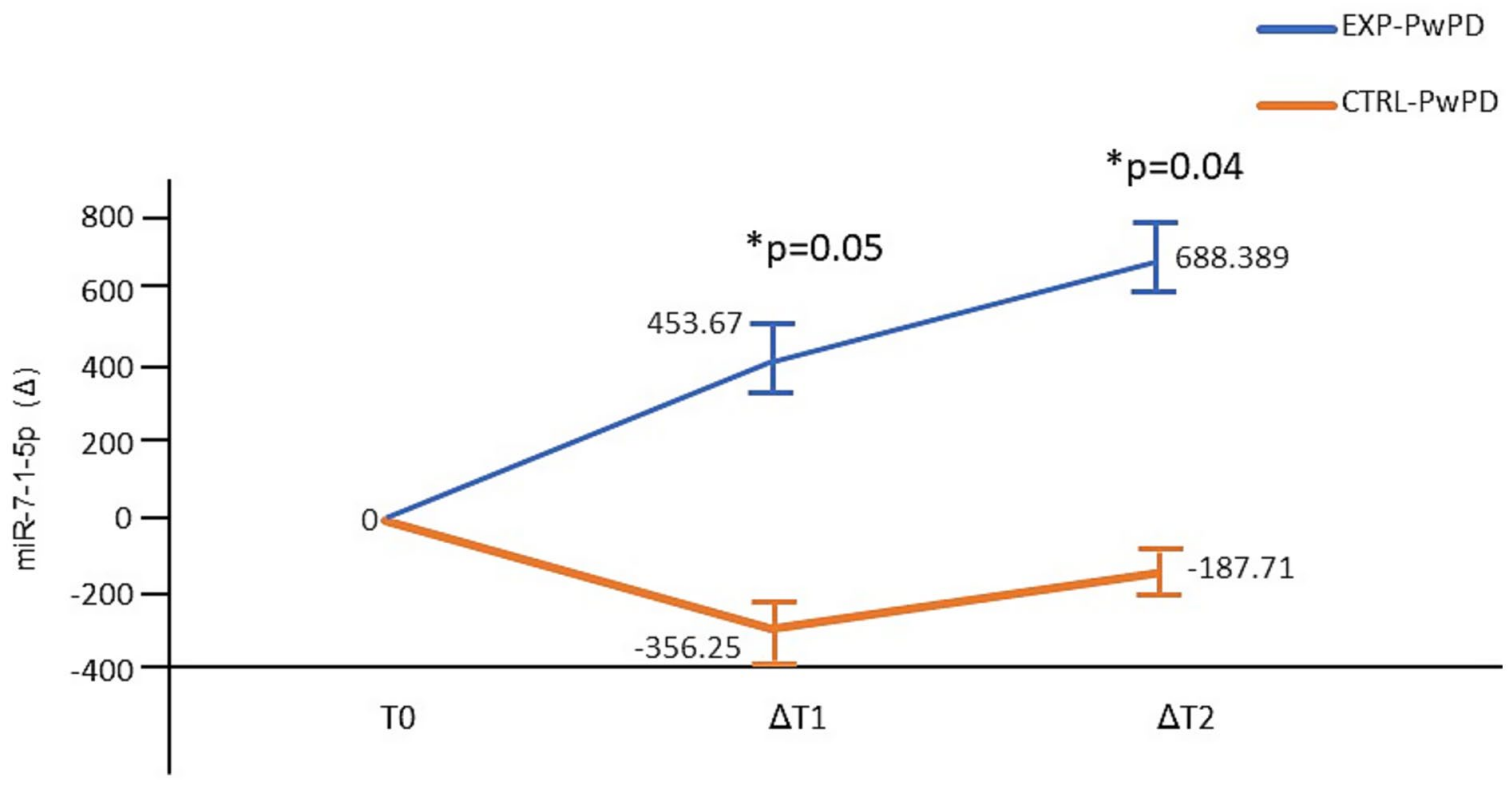
Serum miR-7-1-5p concentration changes from baseline (Δ) at T1 (ΔT1) and T2 (ΔT2) in EXP-PwPD (blue) and CTRL-PwPD (orange) groups. Error bars represent standard deviation. *p*-values indicate the statistical difference of ΔT1 and ΔT2 between EXP-PwPD *vs.* CTRL-PwPD (Student’s *t*-test).

**Figure 6:**
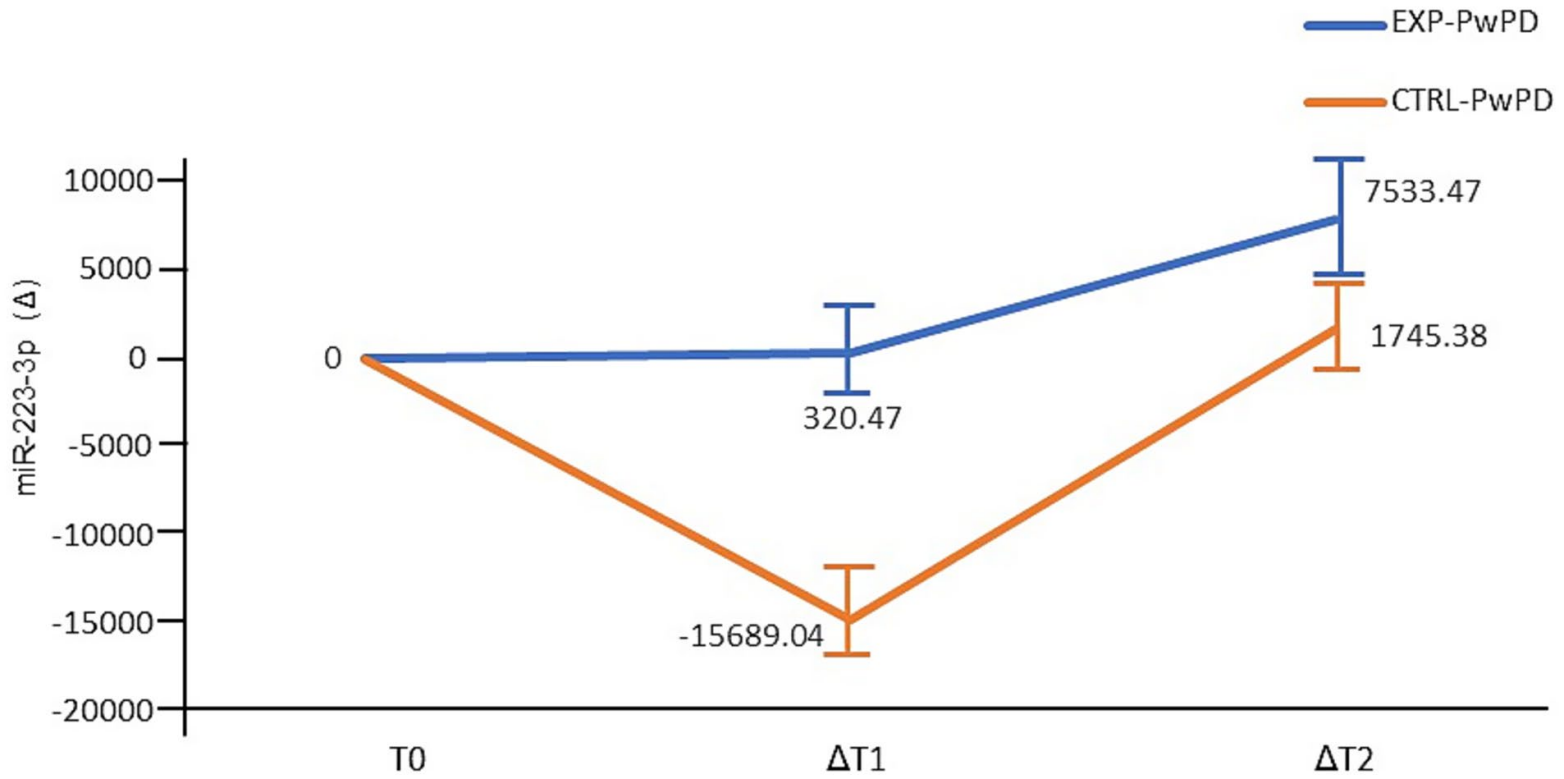
Serum miR-223 concentration changes from baseline (Δ) at T1 (ΔT1) and T2 (ΔT2) in EXP-PwPD (blue) and CTRL-PwPD (orange) groups. Error bars represent standard deviation.

Regarding miRNAs, intensive rehabilitation program was associated with an increase in serum miR-7-1-5p expression at the borderline of statistical significance compared with the home-based rehabilitation program (ΔT1 EXP-PwPD *vs.* CTRL-PwPD: *p* = 0.05; ΔT2 EXP-PwPD *vs.* CTRL-PwPD: *p* = 0.04) (Figure 5). In contrast, no significant statistical differences were observed for serum miR-223-3p concentration (Figure 6).

**Figure 7:**
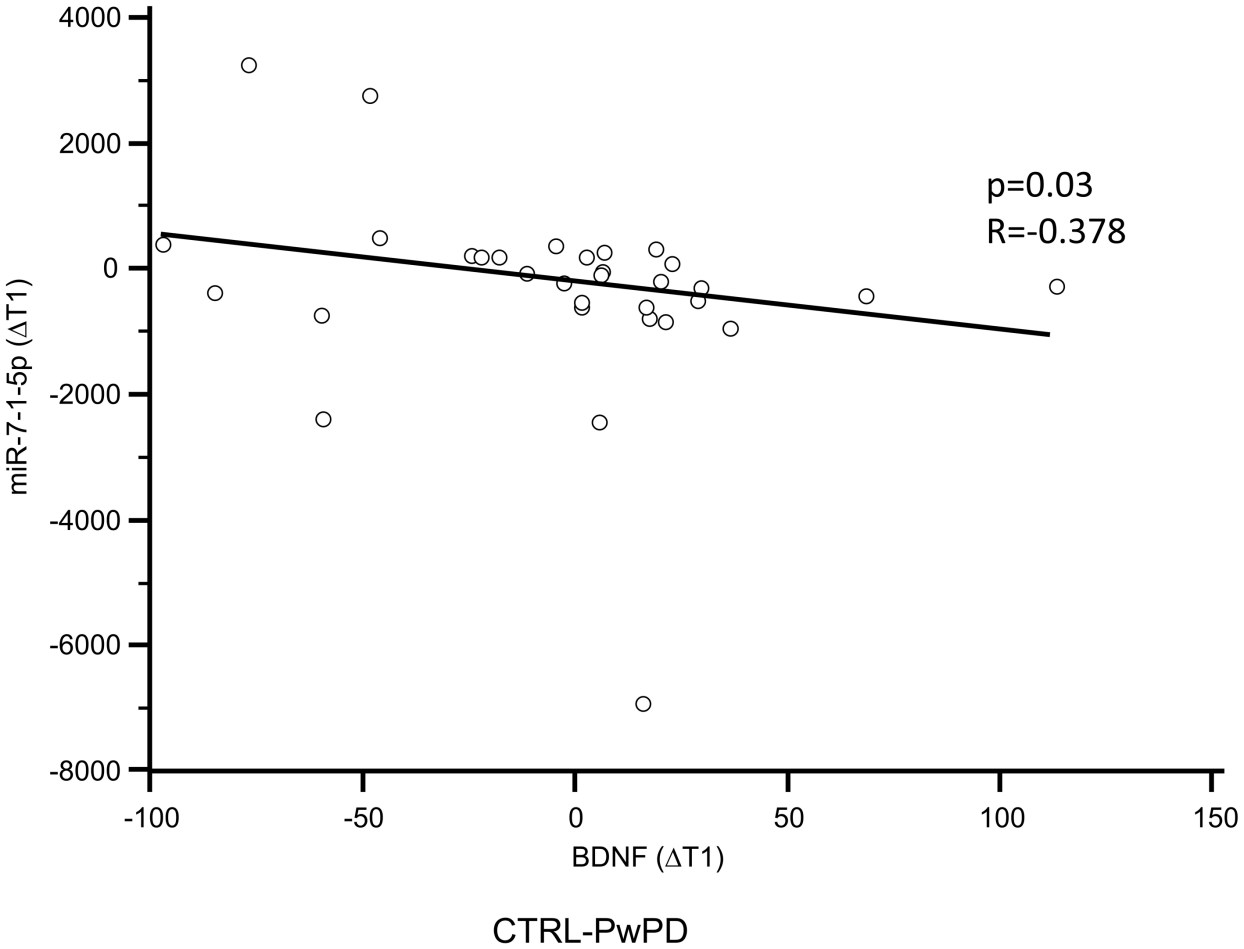
Correlation between serum BDNF and miR-7-1-5p changes from baseline at T1 (∆T1) in CTRL-PwPD group. The *p*-value was calculated by Spearman’s correlation test.

Finally, correlation analyses identified a significant negative correlation between the change in serum concentration of miR-7-1-5p from T0 to T1 (ΔT1) and BDNF concentration in CTRL-PwPD (*p* = 0.03; *R* = −0.378) (Figure 7). This correlation was not observed in EXP-PwPD nor in the overall PwPD cohort. No other significant correlations were found among these four parameters, either at T1 or at baseline (T0). The absolute data of all the analyzed biomarkers for each time point are reported at Table 2.

**Table 2:** Laboratory characteristics of PwPD grouped for rehabilitation program.

Parameters	EXP-PwPD	CTRL-PwPD
BDNF (ng/ml) T0	70.22; 17.51–90.60	81.49; 45.71–98.27
BDNF (ng/ml) T1	76.28; 44.69–102.02	74.25; 38.21–90.33
BDNF (ng/ml) T2	84.18; 30.87–105.72	67.52; 55.54–76.56
α-syn (ng/ml) T0	13.30; 8.71–17.36	13.68; 8.20–22.65
α-syn (ng/ml) T1	18.54; 12.71–26.23	13.30; 7.95–21.15
α-syn (ng/ml) T2	11.87; 9.08–28.59	13.29; 7.96–19.93
miR-7-1-5p (copies/ng) T0	1.67×10^2^; 0.01–2.66×10^2^	6.33×10^2^; 2.23×10^2^-9.33×10^2^
miR-7-1-5p (copies/ng) T1	2.67×10^2^; 0.01–8.33×10^2^	2.83×10^2^; 1.30×10^2^-5.67×10^2^
miR-7-1-5p (copies/ng) T2	4.00×10^2^; 1.33×10^2^-8.67×10^2^	3.66×10^2^; 1.77×10^2^-6.67×10^2^
miR-223-3p (copies/ng) T0	1.75×10^4^; 2.91×10^3^-1.25×10^5^	5.05×10^4^; 3.33×10^3^-1.28×10^5^
miR-223-3p (copies/ng) T1	1.99×10^4^; 3.92×10^3^-1.36×10^5^	3.07×10^4^; 2.92×10^3^-2.42×10^5^
miR-223-3p (copies/ng) T2	3.46×10^4^; 3.17×10^3^-1.58×10^5^	1.62×10^4^; 1.58×10^3^-6.00×10^5^

PwPD, Persons with Parkinson’s disease; BDNF, Brain-derived neurotrophic factor; α-syn, alpha synuclein; miR, miRNA. Data are expressed as median; interquartile range (IQR).

## Discussion

This study aimed to explore how an intensive outpatient multidisciplinary rehabilitation program might influence the serum expression of specific PD biomarkers, including two proteins (BDNF and total *α*-synuclein) and two miRNAs (miR-7-1-5p and miR-223-3p), compared to a home-based self-treatment program.

Overall, the two interventions—an outpatient intensive aerobic program and a home-based self-administered stretching routine—elicited distinct biological responses. The intensive rehabilitation program led to an increase in serum expression of total α-synuclein, BDNF, and, to a lesser extent, miR-7-1-5p, while the home-based group did not produce comparable changes for these biomarkers. Interestingly, the timing of these changes varied: total α-synuclein expression increased during the intensive program but returned to baseline after the program ended, whereas the increased concentration of BDNF and miR-7-1-5p persisted for at least 3 months after the program concluded. These findings suggest that exercise intensity influences both the magnitude and persistence of circulating biomarker responses.

### Clinical and rehabilitative perspective

From a clinical perspective, the significant Time × Group interaction indicates that the experimental and control groups followed distinct motor trajectories over the study period. PwPD undergoing intensive multidisciplinary rehabilitation exhibited a marked short-term improvement in motor performance at the end of treatment, whereas no significant changes were observed in the home-based stretching group. When interpreted according to established MCID thresholds for the MDS-UPDRS Part III, the magnitude of motor improvement achieved after intensive rehabilitation reached clear clinical relevance. However, this benefit was not sustained over time, as motor performance progressively returned toward baseline values at the 3-month follow-up. Notably, the deterioration observed between the end of treatment and follow-up exceeded the MCID threshold for clinically important worsening, reflecting a substantial loss of rehabilitation-induced motor gains in the absence of continued intensive training.

In recent years, rehabilitation has gained increasing prominence among clinicians as a key therapeutic component in the management of Parkinson’s disease for various reasons. These include fewer side effects compared to medication and an overall better quality of life (Goldman et al., 2024). Recent long-term studies suggest that regular physical activity could actually modify the progression of PD. A significant prospective study by Tsukita and colleagues (Tsukita et al., 2022) showed that continuing exercise was linked to a slower clinical progression of both motor and non-motor symptoms in patients over time. However, how different rehabilitative approaches influence disease-related biomarkers in body fluids remains insufficiently understood, and direct comparisons between intensive and conventional rehabilitation are still largely lacking (Luthra et al., 2025). Our study contributes to this area by describing differential short and medium-term biological responses associated with rehabilitation intensity.

### Biological perspective

Regarding the biological results, to our knowledge this is the first study to extend monitoring 12 weeks after the end of the program. Our findings show that the intensive exercise approach causes an increase of serum BDNF concentration, confirming that rehabilitation modulates its expression (Frazzitta et al., 2014; Fontanesi et al., 2016; Sajatovic et al., 2017; Landers et al., 2019; O'Callaghan et al., 2020; Szymura et al., 2020; Di Cagno et al., 2023; Harpham et al., 2023; Kaagman et al., 2024). Notably, we found that serum BDNF concentrations remained elevated even 12 weeks after the end of treatment, in contrast to Angelucci et al. (2016) who reported that BDNF increased after 7 days of treatment, but subsequently declined despite the continuation of the intervention and also 30 days post-program. This finding underscores the positive impacts of rigorous exercise for PwPD, which may boost BDNF production in the brain through several mechanisms, including increased blood–brain barrier permeability (Pan et al., 1998) and enhanced dopaminergic signaling in the nigrostriatal pathway (Mitchell et al., 2024).

In parallel, our study found that intensive exercise led to a notable transient increase in total serum *α*-synuclein expression, directly linked to the exercise activities. Once the exercise program paused, the total α-synuclein concentrations returned to baseline expression. This effect was unique to the intensive rehabilitation group, as no changes in α-synuclein concentration were observed in the home-based program. These results are in line with a previous study involving a mouse model of PD (Zhou et al., 2017), which reported higher plasma α-synuclein levels in mice engaged in running wheel exercises compared to control mice. Remarkably, that study and another involving treadmill exercise in mice showed that high-intensity activities decreased α-synuclein expression in the brain (Kumar et al., 2025), suggesting that intense exercise may alleviate symptoms and slow disease progression by reducing abnormal protein accumulation in the brain.

### Biological mechanisms

The mechanisms underlying progressive nigrostriatal neurodegeneration in PD are not fully understood; the biological responses observed in this study may reflect exercise-related adaptive phenomena. *α*-synuclein has been implicated in presynaptic function and synaptic activity, and its peripheral concentration may be influenced by changes in neuronal and systemic activity. Accordingly, high-intensity exercise could plausibly modulate the release and distribution of circulating *α*-syn, potentially contributing to the transient increase observed during intensive rehabilitation, followed by a return toward baseline after training cessation (El-Agnaf et al., 2003; Schapansky et al., 2015; Darragh et al., 2021; Brodin et al., 2022). Alternatively, changes in serum α-syn following intense exercise may be generated by in changes in α-synuclein present outside the central nervous system, in skeletal muscle, platelets, or other blood cells (Nakai et al., 2007). However, further mechanistic and *in vitro* studies are required to clarify these observations and to test this hypothesis.

On the epigenetic front, results herein suggest a trend toward increased miR-7-1-5p concentrations following intensive rehabilitation, while a slight decrease was observed in PwPD following home-based exercise. Similar to the findings with BDNF, the impact of intensive rehabilitation on miR-7-1-5p persisted even 3 months after the program ended. These results align with prior research, noting a decrease in miR-7-1-5p among people engaged in regular exercise (Barber et al., 2019), whereas intensive exercise tends to raise circulating miRNA expression in PwPD (Da Silva et al., 2021). Importantly, this study is the first to evaluate miRNA expression after exercise, discovering that the effects of intensive rehabilitation last for at least 12 weeks.

Although miR-7-1-5p and miR-223-3p generally inhibit BDNF and *α*-synuclein expression, we did not find a negative correlation between these miRNAs and the other two parameters in PwPD at the start of the study. In our earlier research on a different group of PwPD, we also found no association between these circulating miRNAs and *α*-syn (Citterio et al., 2025), suggesting that other pathogenic mechanisms may be involved in the production and modulation of miR-7-1-5p and miR-223-3p. Interestingly, the changes in miR-7-1-5p and BDNF from the beginning to the end of the rehabilitation program showed a negative correlation in the control group, indicating that even mild home-based rehabilitation influences the balance of their serum expressions, restoring their natural negative correlation. This pattern did not occur in the experimental group, possibly because intensive exercise impacts various other pathways where these parameters are involved. The lack of correlation persisted even 12 weeks after the program concluded, which is not surprising given that we observed medium-term effects from intense exercise. In future research, it would be valuable to track miR-7-1-5p and BDNF expression for longer periods after the program ends, such as 6 or even 12 months later, to explore the long-term effects of intensive exercise on these biological markers and proteins.

It is important to note the miR-7-1-5p and BDNF concentration remained elevated for at least 3 months after the program ended, even when clinical motor scores had returned to baseline. In contrast *α*-synuclein serum concentration aligned closely with the motor improvement seen in the experimental group. Thus, *α*-synuclein concentration increased significantly throughout the intensive rehabilitation phase, coinciding with notable improvements in motor skills, and subsequently returned to baseline at follow-up when motor abilities declined. This suggests that the transient elevation of circulating α-synuclein may reflects the heightened neuronal and synaptic activation induced by intensive exercise, rather than representing a causal driver of the observed motor improvement. Thus, the increase in serum α-synuclein appears to be a natural by-product of the enhanced synaptic turnover and vesicular cycling that occur during training. This distinction suggests that intensive rehabilitation triggers a complex biological response: a quick, activity-linked rise of α-synuclein associated with immediate motor enhancement, alongside a longer-lasting neurotrophic and epigenetic response as indicated by the sustained elevation of BDNF and miR-7-1-5p. These enduring molecular changes might represent ongoing neuroplastic or compensatory mechanisms that extend beyond the period of observed motor improvement. However, it is important to interpret the results regarding miR-7-1-5p with caution, as the observed changes were accompanied by *p*-values close to the significance threshold.

This study has several limitations, including a relatively small sample size, which may reduce statistical power and affect the interpretation of the findings, the lack of a healthy subject group, and the absence of mechanistic analyses. Future studies involving larger cohorts, expanded biomarker panels, and extended monitoring are needed to confirm these findings and clarify their functional significance.

## Conclusion

In conclusion, the findings of this study, although preliminary, suggest that in PwPD a highly aerobic and intensive rehabilitation program influences key molecular biomarkers, with more pronounced effects on BDNF, total α-synuclein, and only modest changes in miR-7-1-5p. The coordinated increases in BDNF and miR-7-1-5p, along with the temporary rising of α-synuclein, underscore the existence of exercise-driven molecular plasticity potentially linked to neurotrophic and neuroprotective pathways. These results enhance our understanding that intensive, structured physical rehabilitation not only leads to clinical improvements but may also affect biological pathways relevant to disease progression. Further research integrating molecular, imaging, and clinical outcomes will be essential to validate these effects and develop optimal exercise regimens for maximizing neurobiological benefits.

## Data Availability

The raw data supporting the conclusions of this article will be made available by the authors, without undue reservation.
